# Mutation of Eamy_RS26425, *hldE*, or *pgm* renders *Erwinia amylovora* CFBP 1430 multi-phage resistant and avirulent

**DOI:** 10.1128/aem.01523-25

**Published:** 2026-03-04

**Authors:** Leandra E. Knecht, Yannick Born, Cosima Pelludat, Steven Gayder, Martin J. Loessner, Lars Fieseler

**Affiliations:** 1 Institute of Food and Beverage Innovation, Zurich University of Applied Sciences, Wädenswil, Switzerland; 2 2Institute of Food, Nutrition and Health, ETH Zurich 27219, Zürich, Switzerland; 3 Agroscope, Plant Pathology and Zoology in Fruit and Vegetable Production 419060, Wädenswil, Switzerland; University of Nebraska-Lincoln, Lincoln, Nebraska, USA

**Keywords:** phage, fire blight, *Erwinia amylovora*, bacteriophage

## Abstract

With the rise of antibiotic-resistant pathogens, bacterial infections have become an increasingly significant threat. Bacteriophages have the potential to complement the current treatment options. To ensure that phage biocontrol is efficient and long-lasting, the risk of resistance development must be minimized, and phage resistance mechanisms should be thoroughly investigated. In this study, a Tn5 transposon mutagenesis library of the fire blight pathogen *Erwinia amylovora* was screened to identify multi-phage-resistant mutants. These mutants had transposon insertions in the genes Eamy_RS26425 (formerly Eamy1960, putative NAD-dependent epimerase), *hldE* (putative ADP-heptose synthase), or *pgm* (putative phosphoglucomutase) and were able to survive attacks of phages that require different receptors for host infection. The generated knockout mutants were analyzed for modification of outer membrane structures and for potential fitness loss. Indeed, multiple alterations that could explain phage resistance were observed. Lipopolysaccharide structures were altered in all three deletion mutants. Amylovoran production was diminished, or almost absent, and cellulose production was affected in the deletion mutants. These findings strongly indicate that the affected enzymes are essential for the correct synthesis of polysaccharides that are targeted by phages as receptors. However, modification of these genes as a resistance strategy against phage attack does not pose a risk to phage treatment, since deletion mutants demonstrated reduced virulence on detached blossoms.

**IMPORTANCE:**

The understanding of bacteriophage host interactions is essentially needed if bacteriophages are considered as an alternative treatment option for bacterial infections.

## INTRODUCTION

Bacteriophages are viruses that exclusively infect bacteria. With an estimated number of 10^31^ (1–3), they are the most abundant biological entity on earth and outnumber bacteria by at least 10-fold (4). With the rise of antibiotic-resistant bacteria, phage therapy is currently once more highlighted as an alternative treatment option (5). In contrast to antibiotics, phages only recognize and target specific host bacteria, while other and potentially beneficial bacteria are left unharmed (6). Phages do not cause side effects in plants, animals, and humans and are considered environmentally safe (7, 8). Since phages require the host metabolism for replication, phage numbers will increase in the presence of the host bacteria, making the treatment more potent. If host bacteria are absent, phages will eventually decay, for example, through UV irradiation (9, 10). All these abilities make phages a promising agent against pathogenic bacteria. Indeed, efficient phage treatments are already in use in humans, food, and agriculture.

Nevertheless, phage resistance can arise. Phages are highly specialized predators, with an estimated 10^25^ phage infections occurring every second (11). This forces bacteria to adapt and develop resistance mechanisms. It is essential to advance our knowledge of the resistance mechanisms applied by bacteria, to generate efficient phage therapies, and to avoid resistance development that renders the treatment futile. Hence, the lessons learned from antibiotic resistance development should be integrated to generate potent and long-lasting phage treatments that minimize the risk of phage resistance development.

Bacterial resistance mechanisms can target the phage infection at different stages (12, 13). The first point of interaction between phages and potential host cells is the adsorption of the virion to the receptor on the surface of the bacterium and subsequent binding. Bacteria can mutate these receptors or modify their accessibility so that the phage can no longer recognize them as potential host cells (14–16). By combining phages that target different host receptors, the risk of resistance development through receptor alteration could be minimized. Apart from bacterial surface modification, a multitude of different phage resistance mechanisms have been reported (17–26).

In addition, mutation of a global regulatory protein can result in multi-phage-resistant bacteria. The alternative sigma factor RpoN was previously identified to provide cross-resistance against phages targeting different host receptors when mutated (27). Global regulators are likely to control the expression of several phage receptors simultaneously and can therefore affect the infectivity of different phages. The danger these global regulators pose to phage therapy is yet to be discovered. In general, careful and thorough investigation of the bacterial host is most advantageous. Identifying and investigating phages that are known to have the ability to bypass or outwit the applied resistance mechanism should further help to minimize the risk of resistance development.

Phage therapy could be a useful solution for the plant pathogen *Erwinia amylovora*, the causative agent of fire blight (28). This severe plant disease affects members of the Rosaceae family and was classified as one of the 10 most devastating plant diseases affecting crop production (29, 30). The main route of infection is through the nectarthodes in the blossoms (31). Since the blossoming season spans only several days, it is therefore the most promising stage to manage and prevent the disease. Fire blight outbreaks were successfully treated with antibiotics, but the appearance of antibiotic-resistant strains and increasing public health concerns led to the abandonment of this therapy (32, 33). Phages could be employed as biocontrol agents to manage the disease, and different phage treatments for fire blight are currently under investigation (34–36). An optimal combination of potent phages ensures full infection coverage of *E. amylovora* strains and minimizes the risk of phage resistance development.

The risk of multi-phage resistance in *E. amylovora* through modification of a global regulator is yet to be determined. The only phage receptors that have been experimentally identified are the lipopolysaccharide (LPS), amylovoran, and bacterial cellulose, that is, extracellular polysaccharides (EPS) (37–41).

Phage therapy has the potential to be an effective weapon against pathogenic bacteria. Aside from single phage resistance, multiple phage resistance can render a potent phage cocktail ineffective. To anticipate this risk, genes involved in mediating multiple phage resistance should be identified. In this study, a Tn5 transposon mutagenesis library was screened to identify multi-phage-resistant mutants in *E. amylovora* CFBP1430. Three genes were identified, which generate, when disrupted or deleted, multiple phage resistance. The effects of these mutations on the bacterium and on the phages were further investigated. The identified genes, Eamy_RS26425, *hldE*, and *pgm* are involved in LPS and EPS synthesis and account for the observed phage resistance.

## MATERIALS AND METHODS

### Culture conditions

*E. amylovora* strains were cultivated on LB agar at 28°C, *Escherichia coli* strains at 37°C. Kanamycin (50 µg/mL) or ampicillin (100 µg/mL) was added if required. All used and generated strains are summarized in Table 1.

**TABLE 1:** Overview of used and generated bacterial strains, plasmids, and phages

Bacterial strain, plasmid, or phage	Characterization	Reference
*Erwinia amylovora* strains		
CFBP1430	Wild-type strain, isolated in France from *Crataegus* sp	(42)
CFBP1430^SmR^	Streptomycin-resistant CFBP1430 used for knockout generation	(39)
CFBP1430 [pBAD18]	Empty vector control	This work
CFBP1430 ΔRS26425	Deletion of RS26425, replaced with a kanamycin cassette	This work
CFBP1430 ΔRS26425+ RS26425	*in trans* complementation of RS26425 using pBAD18 RS26425	This work
CFBP1430 Δ*hldE*	Deletion of *hldE*, replaced with a kanamycin cassette	This work
CFBP1430 Δ*hldE + hldE*	*in trans* complementation of *hldE* using pBAD18 *hldE*	This work
CFBP1430 Δ*pgm*	Deletion of *pgm*, replaced with a kanamycin cassette	This work
CFBP1430 Δ*pgm + pgm*	*in trans* complementation of *pgm* using pBAD18 *pgm*	This work
4/82	Isolated in Egypt from *Pyrus communis*; low EPS-producer	(42)
*Escherichia coli* strains		
S17-1 λ-pir		(43, 44)
XL1-Blue	Δ(*mcrA*)183 Δ(*mcrCB‐hsdSMR‐mrr*)*173 endA1 supE44 thi‐1 recA1 gyrA96* *relA1 lac* [F' *proAB lacI*^q^*ZΔM15* Tn*10* (Tet^r^)]	NEB (Ipswich, USA)
Dh5α	supE44 ∆lacU169 (φ80lacZ∆M15) hsdR17 recA1 endA1 gyrA96, thi-1 relA1	(45)
Plasmids		
pSB315	Containing kanamycin cassette without transcriptional terminator, Amp^R^, Kan^R^	(46)
pKAS32	Suicide vector with *rpsL* gene, Amp^R^	(47)
pKAS32 ΔRS26425	Deletion vector exchanging RS26425 with a kanamycin cassette	This work
pKAS32 Δ*hldE*	Deletion vector exchanging *rfaE* with a kanamycin cassette	This work
pKAS32 Δ*pgm*	Deletion vector exchanging *pgm* with a kanamycin cassette	This work
pBAD18	Complementation vector, arabinose-induced pBAD promoter	(48)
pBAD18 RS26425	Complementation vector for *topB1*, artificial RBS	This work
pBAD18 *hldE*	Complementation vector for *rfaE*, artificial RBS	This work
pBAD18 *pgm*	Complementation vector for *pgm*, artificial RBS	This work
Phages		
Bue1	*Ackermannviridae*, Nezavisimistyvirus	(38)
L1	*Autotranscriptaviridae*, Elunavirus	(49)
M7	*Andersonviridae*, Kolesnikvirus	(49)
S2	*Autosignataviridae*, Eracentumvirus	(38)
S6	*Schitoviridae*, Waedenswilvirus	(49)
Y2	*Chaseviridae*, Loessnervirus	(49)

### Soft agar overlay and phage propagation

Phages were propagated using the soft agar overlay method (50). Molten LB+ soft agar (LB broth, 4 g/L agar, 2 mM MgSO_4_, 10 mM CaCl_2_) was supplemented with 90 µL of an overnight bacterial suspension and 10 µL of a phage suspension. Soft agar was then evenly spread onto LB plates to generate semi-confluent lysed plates. The plates were incubated overnight before 5 mL SM buffer (100 mM NaCl, 8 mM MgSO_4_, 50 mM Tris-Cl, pH 7.4) per plate was added, and the plates were incubated for 5 h at room temperature (RT) under shaking. NaCl was added (0.5 M) to the supernatant, and the mixtures were incubated for 30 min at RT before centrifugation (10 min, 10,000 × *g*). Phages in the supernatant were then polyethylene glycol treated (10% wt/vol PEG8000, ice bath overnight), spun down (15 min, 10,000 × *g*, 4°C), CsCl density gradient purified (51), and dialyzed three times against SM buffer (Bue1, L1, M7, S2, and S6). In the case of Y2, PEG was removed by incubating the PEG-phage mixture at RT for 1 h. The solution was then centrifuged at 5,000 × *g* for 10 min. Phages in the supernatant were sterile filtered (0.22 µm filter). The phages were stored at 4°C (Table 1).

### Screening of the library

To identify mutants resistant against a collection of phages, a Tn5 transposon mutagenesis library was generated as described previously (39). Overnight cultures of the transposon library were prepared using a 96-well replica stamp. Molten LB+ soft agar was supplemented with kanamycin (25 µg/mL) and 5% glycerol. Of each plate, two replicates were generated: one supplemented with phages Bue1 (10^8^ PFU/mL), L1 (10^7^ PFU/mL), M7 (10^6^ PFU/mL), S2 (10^7^ PFU/mL), S6 (10^5^ PFU/mL), or Y2 (10^5^ PFU/mL), the other plate without phages in the soft agar. A total of 200 µL soft agar per well was distributed into a 96-well flat-bottom plate. Immediately after distribution, the bacterial starter culture was transferred into the prepared soft agar plate using a 96-well replica stamp. The plates were incubated overnight at 25° (S6) or 28°C (Bue1, L1, M7, S2, and Y2) for optimal phage infection, and the optical density of each well was measured using a Synergy H1 Multi-Mode Microplate Reader (BioTek Instruments, Inc.). The difference in optical density of the grown bacterial cultures inoculated with and without phages was calculated to identify phage-resistant mutants. Phage-insensitive mutants grow similarly in the absence and presence of phages, resulting in a low difference. Mutants remaining sensitive to phages grow weaker in the presence of the phages; therefore, the calculated difference in optical density is larger. Transposon mutants able to grow in the presence of phages to comparable optical densities as in the absence were validated in soft agar overlays for plaque formation.

### Identification of Tn5 insertion sites in the *E. amylovora* genome

To identify insertion sites of the Tn5 transposon, an arbitrary-primed PCR as described by Das et al. (52) was performed (52). The used primers are listed in Table 2. Arbitrary primers Arb-P1, Arb-P2, and Arb-P3 were paired with the first nested primer. Using the purified PCR product as a template, the anchor primer and the second nested primer amplified a shorter segment, which was purified and sequenced (Microsynth, Switzerland). The sequences were compared to the *E. amylovora* CFBP1430 genome (accession number FN434113) to locate the transposon insertion site.

**TABLE 2:** Oligonucleotides used in this study

Primer	Sequence 5'−3'	Characterization	Reference
Arb-P1	GGCCACGCGTGCACTAGTCANNNNNNNNNNGCTCG	Arbitrary PCR step 1	(52)
Arb-P2	GGCCACGCGTGCACTAGTCANNNNNNNNNNGACTC	Arbitrary PCR step 1	(52)
Arb-P3	GGCCACGCGTGCACTAGTCANNNNNNNNNNGATAC	Arbitrary PCR step 1	(52)
Kan-2-FP	ACCTACAACAAAGCTCTCATCAACC	Arbitrary PCR step 1	episome Tn5 <Kan2>
Anchor-P	GGCCACGCGTGCACTAGTCA	Arbitrary PCR step 2	(52)
Nested f	TTCAGGGTTGAGATGTGTATAAGAGACAG	Arbitrary PCR step 2	This work
pSB315 f	GAAAGCCACGTTGTGTCTC	Kanamycin cassette	(46)
pSB315 r	CCTTCATTACAGAAACGGC	Kanamycin cassette	(46)
pBAD f	CTGTTTCTCCATACCCGT T	Plasmid insert	
pBAD r	CTCATCCGCCAAAACAG	Plasmid insert	
pKAS32-RS26425_up-fw	CAGATCTGCGCGCGATCGATGCGCTTATCTCTGCAAACAG	CFBP1430 ΔRS26425	This work
pKAS32-RS26425_up-rev	GAGTTTTTCTAATGGCAGACTGATGGCCTGAAAAAAAAG	CFBP1430 ΔRS26425	This work
pKAS32-RS26425_kan-fw	TCAGTCTGCCATTAGAAAAACTCATCGAGC	CFBP1430 ΔRS26425	This work
pKAS32-RS26425_kan-rev	CGAGGGCACTATGAGCCATATTCAACGG	CFBP1430 ΔRS26425	This work
pKAS32-RS26425_down-fw	TATGGCTCATAGTGCCCTCGTTAATTATGCAATG	CFBP1430 ΔRS26425	This work
pKAS32-RS26425_down-rev	CGCAAATTTAAAGCGCTGATCCACCGTTCCGGTCGGTA	CFBP1430 ΔRS26425	This work
pBAD-RS26425-fw	TGGGCTAGCGAATTCGAGCTCAGGAGGTTCGTATGAAATATCTGGTCACC	Complementation CFBP1430 ΔRS26425	This work
pBAD-RS26425-rev	TGCATGCCTGCAGGTCGACTCTAGATTACTTGTGATAAAACTCTTTATAC	Complementation CFBP1430 ΔRS26425	This work
pKAS32-hldE_up-fw	TGCGCATGCTAGCTATAGTTCTAGATAATGAAGGTCAGCGATC	CFBP1430 Δ*hldD*	This work
pKAS32-hldE_up-rev	TATGGCTCATTCCAGAGACTCCAGACAG	CFBP1430 Δ*hldD*	This work
pKAS32-hldE_kan-fw	AGTCTCTGGAATGAGCCATATTCAACGG	CFBP1430 Δ*hldD*	This work
pKAS32-hldE_kan-rev	CCGTAATCCGTTAGAAAAACTCATCGAGC	CFBP1430 Δ*hldD*	This work
pKAS32-hldE_down-fw	GTTTTTCTAACGGATTACGGCTGCCCGC	CFBP1430 Δ*hldD*	This work
pKAS32-hldE_down-rev	TGGAATTTCCCGGGAGAGCTCGTTGTTCTGCACCATCATGGGC	CFBP1430 Δ*hldD*	This work
pBAD-hldE-fw	TGGGCTAGCGAATTCGAGCTCTGGAGTCTCTGGAATGAAAATTAC	Complementation CFBP1430 Δ*hldD*	This work
pBAD-hldE-rev	TGCATGCCTGCAGGTCGACTCTAGATCAGTTTTGCCCGGTTCC	Complementation CFBP1430 Δ*hldD*	This work
pKAS32-pgm_up-fw	TGCGCATGCTAGCTATAGTTCTAGATATCCTGGGCCATTAGCGG	CFBP1430 Δ*pgm*	This work
pKAS32-pgm_up-rev	TATGGCTCATTGGCGCTTCTCCCTGACG	CFBP1430 Δ*pgm*	This work
pKAS32-pgm_kan-fw	AGAAGCGCCAATGAGCCATATTCAACGG	CFBP1430 Δ*pgm*	This work
pKAS32-pgm_kan-rev	GCCGGTCAAATTAGAAAAACTCATCGAGC	CFBP1430 Δ*pgm*	This work
pKAS32-pgm_down-fw	GTTTTTCTAATTTGACCGGCGGTGAAAAG	CFBP1430 Δ*pgm*	This work
pKAS32-pgm_down-rev	TGGAATTTCCCGGGAGAGCTCATAATGTTGATAAAGACCCATACGTC	CFBP1430 Δ*pgm*	This work
pBAD-pgm-fw	TGGGCTAGCGAATTCGAGCTCAGGGAGAAGCGCCAATGG	Complementation CFBP1430 Δ*pgm*	This work
pBAD-pgm-rev	TGCATGCCTGCAGGTCGACTCTAGATCAGGCGTTATTCAGAACCG	Complementation CFBP1430 Δ*pgm*	This work

### Generation of knockout mutants

The Tn5 library screen revealed mutants with transposon insertions into the genes *hldE*, *pgm,* and Eamy_RS26425 to be resistant against multiple phages. To investigate the impact of these genes on the phage infection process, knockout mutants were generated (Tables 1 and 2). The gene deletions were generated by allelic exchange using the suicide plasmid pKAS32 carrying an R6K origin of replication (47). Flanking regions (approx. 1,000 bp upstream and downstream) of the genes of interest and a kanamycin cassette (*aphT*) amplified from pSB315 (53) were integrated into the multiple cloning site of pKAS32. The three fragments were amplified using Gibson assembly primers designed by the NEBuilder tool (NEBuilder Assembly Tool v1.12.18) using the KAPA HIFITM PCR kit (KAPA Biosystems, Wilmington, USA). The fragments were joined by overlap extension PCR. The vector pKAS32 was extracted from *E. coli* S17-1 λpir (pKAS32) using the NucleoSpin Plasmid Kit (Macherey-Nagel; Düren, Germany) and linearized by SacI and XbaI. The product was analyzed by gel electrophoresis and purified from a 1% agarose gel by the DNA Clean & Concentrator−5 Kit by Zymo Research. The suicide plasmid was generated either through Gibson assembly (54) or through classic ligation using a T4 polynucleotide ligase (Thermo Fisher Scientific) according to the manufacturer’s instructions. The plasmids were introduced into electrocompetent, streptomycin-resistant *E. amylovora* CFBP1430 (39) by electroporation. Cells were resuspended in SOC and incubated at 30°C with vigorous shaking for 1 h. The cells were then plated on LB plates amended with kanamycin and streptomycin to select for deletion mutants. Alternatively, the deletion vector was introduced by mating. Overnight cultures of *E. amylovora* and *E. coli* S17-1 λ-pir containing the deletion vector were grown in LB containing streptomycin (100 µg/mL) or ampicillin. Bacteria were washed twice in PBS buffer and mixed at different ratios (*E. coli:E. amylovora* 1:10, 1:100). Cells were then centrifuged (5,000 × *g*, 2 min), and the pellet was spotted onto LB plates and incubated at 37°C. After 3, 6, and 24 h, cells were scraped off and plated onto MM2 plates supplemented with kanamycin and streptomycin to select the correct mutants. Correctness of deletion was checked by PCR.

### Complementation of knockout mutants

The arabinose-inducible plasmid pBAD18 (48) holding an ampicillin resistance gene was linearized with SacI and XbaI, and purified. The inserts and their ribosomal-binding sites were amplified from *E. amylovora* CFBP1430 by PCR with Gibson primers (NEB). PCR products were recovered from a 1% agarose gel. The linearized pBAD18 vector and the insert were joined by Gibson assembly (54). The plasmids were then introduced into electrocompetent *E. coli* XL1-Blue cells for amplification. Cells were recovered in SOC and incubated for 1 h at 37°C with vigorous shaking before plating onto LB plates containing ampicillin. Correct insertion was verified by PCR. Correct plasmids were extracted and introduced into electrocompetent *E. amylovora* CFBP1430 knockout mutants. Phage resistance of the generated mutants was monitored using the spot on the lawn technique with dilutions of Bue1, L1, M7, S2, S6, and Y2.

### *In vitro* infection assay

The infectivity of the six phages toward the generated mutants was tested. *In vitro* infections were carried out to monitor the growth of the mutants in the presence of phages. *E. amylovora* CFBP1430 and the generated mutants were grown overnight in LB medium. Cells were then washed twice in LB+ medium, and OD_600nm_ was adjusted to 10^7^ CFU/mL. A total of 20 µL per sample was transferred to 1,960 µL LB and supplemented with 20 µL phage (10^10^ PFU/mL). Mixtures were then distributed into a 96-well flat-bottom plate. The samples were incubated at 25°C with double orbital shaking in a plate reader for 40 h. OD_600nm_ was measured every half hour.

### Adsorption of phages to the cell wall

Receptor-binding affinity of the generated mutants was tested using adsorption experiments (37). Overnight cultures of the mutants were washed with LB medium twice and diluted to an OD_600nm_ of 1.0. A total of 990 µL per sample was transferred to 2 mL test tubes, and 10 µL Bue1, L1, S2, or Y2 (10^9^ PFU/mL) were added and incubated for 10 min at RT under shaking. Samples were then centrifuged at 10,000 × *g* for 10 min at 4°C. Unbound phages in the supernatant were quantified using the soft agar overlay method, plating on *E. amylovora* CFBP1430. As controls, phages were incubated with wild-type (wt) bacteria or medium only. The phages M7 and S6 were excluded from the experiment since previous experiments have demonstrated poor reproducibility (own observation, unpublished).

### Amylovoran measurement

The impact of *hldE*, *pgm,* and RS26425 deletions on amylovoran synthesis was analyzed using the amylovoran-cetylpyridinium chloride (CPC) precipitation assay (55). The generated mutants and the wt were grown in MM2 (50) at 28°C under shaking for 24 h. The samples were adjusted to an OD_600nm_ of 1.0. A total of 1 mL per sample was centrifuged for 5 min at 10,000 × *g,* and 950 µL supernatant per sample was mixed with 50 µL CPC (50 mg/mL). After incubating the samples for 10 min at RT, the OD_600 nm_ of each sample was measured. The low EPS-producing strain *E. amylovora* 4/82 was used as a control.

### LPS silver staining

Potential alterations in the LPS structure were analyzed by silver staining. Cells were grown in LB and washed twice in PBS buffer before the OD_600nm_ was adjusted to 1.0, and 1 mL per sample was centrifuged at 8,000 × *g* for 5 min. The pellet was resuspended in 100 µL SDS sample buffer (90 mM Tris base, 2% SDS, 0.02% Bromophenol blue, 20% sucrose, pH adjusted to 6.8 in H_2_O) and boiled for 10 min at 100°C. After cooling the samples to RT, 2.5 µL of proteinase K (20 µg/µL) was added, and samples were incubated for 1 h at 60°C. The samples were subsequently loaded onto an SDS PAGE gel (12% resolving/4% stacking gel) and let run with 35 A for 2 h. The gels were washed in ddH_2_O before being soaked with fresh fixing solution (40% ethanol and 5% acetic acid in H_2_O) for 1 h. The fixing solution was subsequently replaced with fresh oxidizing solution (fixing solution supplemented with 30 mM periodic acid) for 5 min. Oxidizing solution was removed, and the gels were washed 3–5 times with at least 500 mL of ddH_2_O for 15 min to completely remove the oxidizing solution. The gels were soaked in freshly prepared staining solution (1.5 mL ammonium hydroxide solution 33%, 14 mM NaOH solution, 0.5% AgNO_3_ in 200 mL H_2_O) for 15 min. After washing the gels several times in ddH_2_O, the gels were developed with freshly prepared developer solution (200 mL ddH_2_O supplemented with 50 mg citric acid and 100 µL formaldehyde solution) until bands appeared. The development was stopped with several charges of ddH_2_O.

### Cellulose visualization

To investigate alterations in the cellulose production provoked by the gene modifications, qualitative cellulose visualization was performed. LB agar without NaCl was supplemented with 40 μg/mL Congo Red and 20 μg/mL Coomassie Brilliant Blue before pouring. Ampicillin and/or 0.2% arabinose were added if required. Per sample, 5 µL was spotted onto plates and incubated for 2 days at RT. Cellulose-producing bacteria appear as pink colonies, while non-producing bacteria remain white (39).

### Growth curves

The impact of the mutations on the fitness of the generated strains was investigated. Bacteria were washed twice in SM buffer, and the OD_600 nm_ was adjusted to 0.1. Cells were diluted in LB medium supplemented with MgSO_4_ (2 mM) and CaCl_2_ (10 mM) or MM2 medium (50) to a concentration of 10^5^ CFU/mL. Cultures were incubated at 28°C for 24 h with double orbital shaking (150 rpm) in a plate reader.

### Detached flower assay

Virulence of the generated *E. amylovora* mutants was tested by a detached flower assay using fresh blossoms from 2-year-old Golden Delicious apple trees (56). Blossoms were treated with *E. amylovora* CFBP1430, the knockout mutants, or PBS buffer (3 mM KCl, 137 mM NaCl, 2 mM KH_2_PO_4_, 10 mM Na_2_HPO_4_) as mock infection. Racks were autoclaved before the experiment, and 24 alternating wells per rack were filled with 2 ml H_2_O. The wells were sealed with perforated scotch tape. Stems were cut freshly to ensure water uptake before transferring the blossoms into the filled wells. Bacteria grown overnight on plates were carefully scratched off and resuspended in PBS, and the optical density was adjusted to 1.0. Subsequently, a 1:50 dilution was performed to generate approximately 10^7^ CFU/mL. A total of 20 µL per treatment was pipetted directly onto the receptacle. The racks were placed into storage boxes (5 L), laid out with paper towels, and soaked with 100 mL H_2_O to ensure humidity. Boxes were incubated at 26°C for 4–5 days before the readout, according to an adjusted rating system, was carried out (57). Healthy blossoms without disease symptoms are classified as grade 1. Visible symptoms on the blossom (browning of the calyx) are referred to as grade 2. Blossoms with disease symptoms in the calyx and the stipe of the blossoms correspond to grade 3.

## RESULTS

### Screen identifies Eamy_RS26425, *hldE,* and *pgm*

The screen was carried out for six phylogenetically diverse phages classified as Nezavisimistyvirus Bue1 (LPS dependent, own observation, unpublished) (38), Elunavirus L1 (amylovoran dependent) (37, 49), Kolesnikvirus M7 (cellulose dependent, own observation, unpublished) (49), Eracentumvirus S2 (amylovoran dependent, own observation, unpublished) (38), Waedenswilvirus S6 (cellulose dependent) (39, 49), and Loessnervirus Y2 (LPS specific) (40, 49) (Table 1). Mutants with transposon insertions into Eamy_RS26425, *hldE,* and *pgm* were observed to be able to resist infection of multiple phages simultaneously. One mutant with an insertion into *hldE* was identified as L1, M7, S2, S6, and Y2 resistant. Only the phage Bue1 was observed to lyse the transposon-disrupted *hldE* mutant. The *hldE* gene is predicted to encode an ADP-heptose synthase. In *E. coli* K12, the homologous bifunctional protein HldE catalyzes the phosphorylation of D-*glycero*-D-*manno*-heptose-7-phosphate to form D-*glycero*-β-D-*manno*-heptose-1,7-bisphosphate and the ADP transfer from ATP to D-*glycero*-β-D-*manno*-heptose-1-phosphate, generating ADP-D-*glycero*-β-D-*manno*-heptose, a precursor for LPS inner core heptoses (58).

The gene *pgm* was identified to be involved in phage susceptibility. As the *hldE* transposon mutant, the *pgm* disrupted mutant was observed to be insensitive against L1, M7, S2, S6, and Y2, but not Bue1. The *pgm* gene is annotated to encode a phosphoglucomutase, an enzyme involved in the breakdown and synthesis of glucose (59).

A total of eight mutants with independent transposon insertions into the gene Eamy_RS26425 were identified during the screen. Phage resistance for these mutants was observed against Bue1, Y2, M7, and S6. The phage S2 was still able to lyse all the tested Eamy_RS26425 transposon mutants. L1 was unable to infect one of these mutants but was observed to lyse the other seven mutants. The Eamy_RS26425 gene is annotated to encode an NAD-dependent epimerase. These proteins use nucleotide-sugar substrates for different chemical processes with NAD as cofactor (60). The Eamy_RS26425 is part of a small, so far uncharacterized operon consisting of four genes: *rssB*, *galU*, Eamy_RS26430, and Eamy_RS26425. The *rssB* gene likely encodes a two-component system response regulator, *galU* an UTP-glucose-1-phosphate uridylyltransferase, and Eamy_RS26430 an UDP-glucose dehydrogenase. The precise function of these genes remains unknown.

To exclude possible downstream effects of the transposon insertions, knockout mutants were generated. To verify whether the gene deletions still had an impact on phage infectivity, resistance was tested in soft agar overlays. The deletion of *hldE* was observed to mediate resistance against Bue1, Y2, M7, S6, and S2. The phage L1 was shown to be unaffected by the *hldE* deletion. Mutants with a deletion of the phosphoglucomutase encoding gene *pgm* were able to resist infection by all phages with the exception of M7. Removing the gene Eamy_RS26425 rendered the mutant resistant against Bue1, Y2, M7, and S6. Both L1 and S2 were still able to lyse the Eamy_RS26425 mutant. Complementation of the three genes restored phage infectivity when arabinose was added to the medium to induce the promoter. In certain cases, the complementation already restored the phage-sensitive phenotype in the absence of arabinose, likely due to the minor leakage of the arabinose promoter (Table 3). Notably, the addition of arabinose to the ∆*pgm* mutant restored infectivity of Bue1, L1, and S2, while the addition of galactose restored infectivity of all phages tested. The addition of other plant-derived carbohydrates such as fructose, glucose, and sucrose did not reveal the effect (Table 4).

**TABLE 3:** Infection of the generated mutants by the six different phages^*a*^

	wt		ΔRS26425		Δ*hldE*		Δ*pgm*		Vector control		ΔRS26425 + RS 26425		Δ*hldE + hldE*		Δ*pgm* + *pgm*	
	–	Ara	–	Ara	–	Ara	–	Ara	–	Ara	–	Ara	–	Ara	–	Ara
Bue 1	+	+	−	−	−	−	−	+	+	+	+	+	+	+	+	+
L1	+	+	+	+	+	+	−	+	+	+	+	+	+	+	−	+
M7	+	+	−	−	−	−	+	+	+	+	+	+	+	+	+	+
S2	+	+	+	+	−	−	−	+	+	+	+	+	−	+	−	+
S6	+	+	−	−	−	−	−	−	+	+	+	+	+	+	−	+
Y2	+	+	−	−	−	−	−	−	+	+	+	+	−	+	−	+

^*a*^ Spot on the lawn experiments were carried out on LB plates. Arabinose (0.2% wt/vol) was added as a control and to induce the promoter of the complementants. As further controls, the wt and the empty vector control were used. Successful infection is depicted by +, whereas − represents lack of infection and plaques. Ara: arabinose.

**TABLE 4:** Infection of the Δ*pgm* mutant by the six different phages in the presence of different monosaccharides and sucrose (each 0.2% wt/vol)^*a*^

	Δ*pgm*					
	–	Ara	Fru	Gal	Glu	Suc
Bue 1	−	+	−	+	−	−
L1	−	+	−	+	−	−
M7	+	+	−	+	−	−
S2	−	+	−	+	−	−
S6	−	−	−	+	−	−
Y2	−	−	−	+	−	−

^*a*^ Successful infection is depicted by +, whereas − represents lack of infection and plaques. Ara: arabinose; Fru: fructose; Gal: galactose; Glu: glucose; Suc: sucrose.

### Reduced phage adsorption in the generated mutants

**Fig 1:**
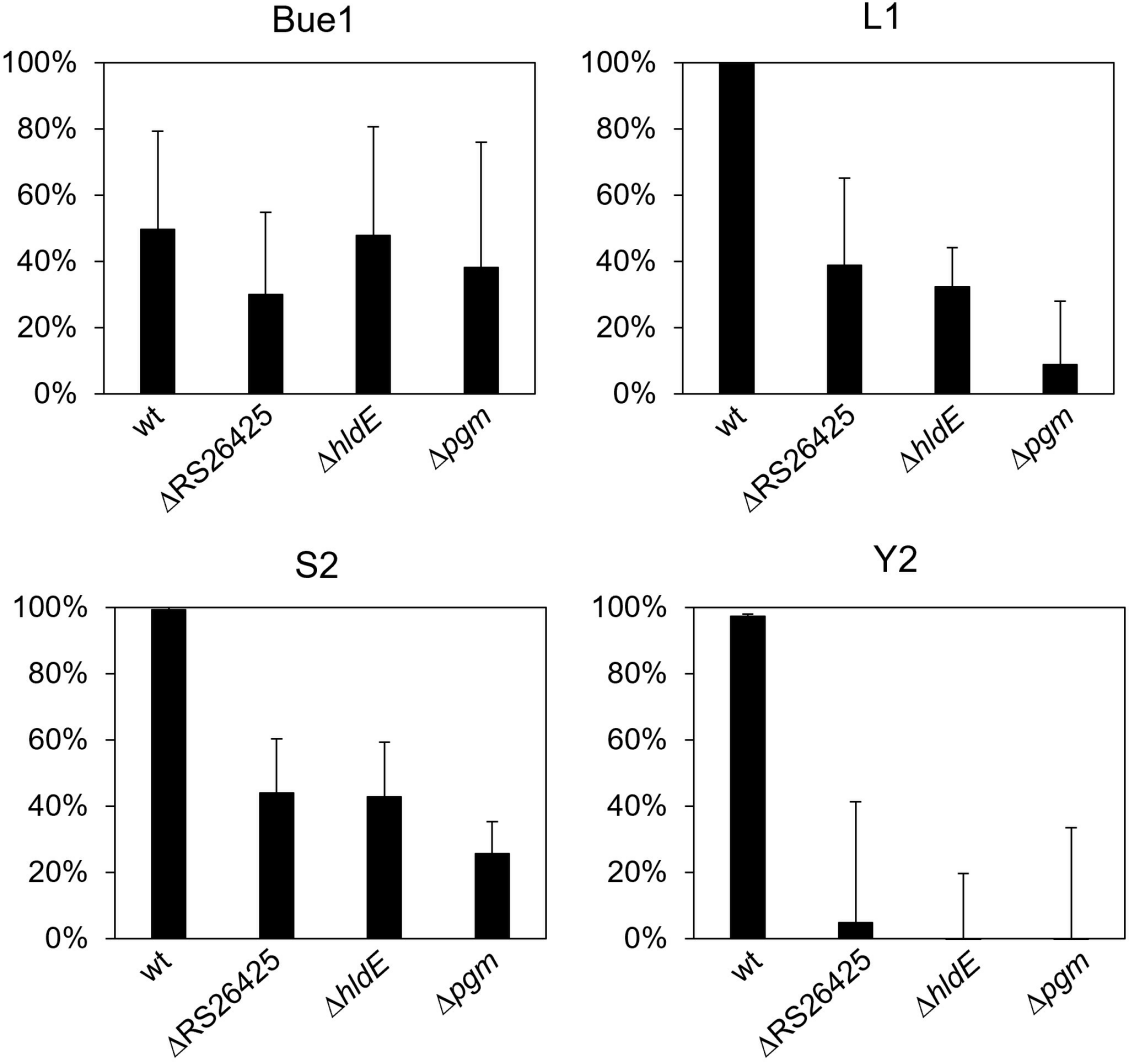
Adsorption of Bue1, L1, S2, and Y2 to the generated mutants. The ability of these four phages to bind to the different mutants was tested. Each sample was tested at least three times independently. Error bars indicate standard deviation.

To identify how the generated mutants affect the six different phages, different experiments were carried out. Adsorption experiments for Bue1, L2, S2, and Y2 were performed to verify whether adsorption to the generated deletion mutants was modified. As illustrated in Fig. 1, Y2 was unable to adsorb to all three deletion mutants. Thus, lack of recognition and binding accounts for the inability of Y2 to infect the mutants. L1 and S2 were both still able to bind to the generated mutants. However, binding affinity was significantly reduced compared to the wt. L1 and S2 adsorption to Δ*pgm* was observed to be the weakest of all three tested mutants. In contrast to the other phages, Bue1 generally showed intermediate adsorption with strong variation. The phages M7 and S6 generated no reproducible data in the experiments and were therefore excluded.

### Alterations in LPS of all three deletion mutants

**Fig 2:**
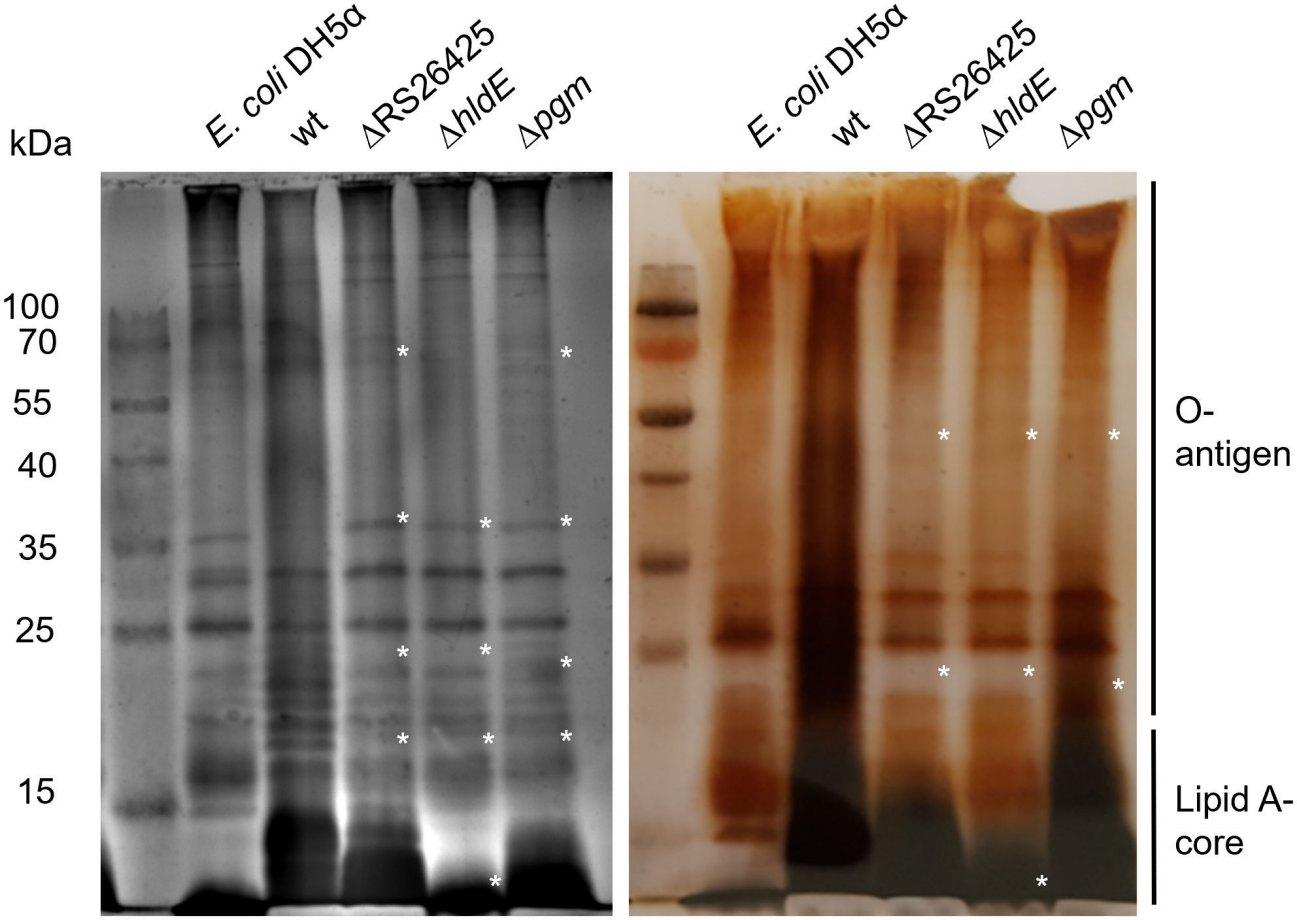
Silver staining of extracted LPS. ΔEamy_RS26425, Δ*hldE*, and Δ*pgm* exhibited alterations of the LPS banding pattern compared to the wt. *E. coli* DH5α was used as an additional control. The left lane was loaded with a protein size marker. The size of the marker proteins is indicated on the left. The image of the left gel was taken by an Azure Biosystems C300, whereas the image on the right was taken by a standard photo camera. Stars indicate alterations in the band pattern compared to the wt. kDa: kilo Dalton.

The binding affinity of Y2 toward the knockout mutants was observed to be abolished. Since Y2 was suggested to recognize LPS structures on the host surface (40), the mutants’ LPS was analyzed for possible modifications. Alterations were observed for all three knockout mutants (Fig. 2). The Eamy_RS26425 deletion mutant lacks a total of four bands between 15 and 25 kDa and possibly at 40 kDa. The deletion of *hldE* generated a strong alteration of the Lipid A and the core region. In addition, three bands between 15 and 25 kDa and possibly bands at 40 and 70 kDa are absent in the LPS pattern of Δ*hldE* compared to the wt. The mutant lacking the gene *pgm* was observed to have the closest LPS pattern compared to the wt. As for the other two mutants, a band at ca. 18 kDa is missing, and bands are more prominent between 55 and 70 kDa. In general, the band at 35 kDa appears much stronger for all three mutants compared to the wt.

### Amylovoran production is affected in mutants

**Fig 3:**
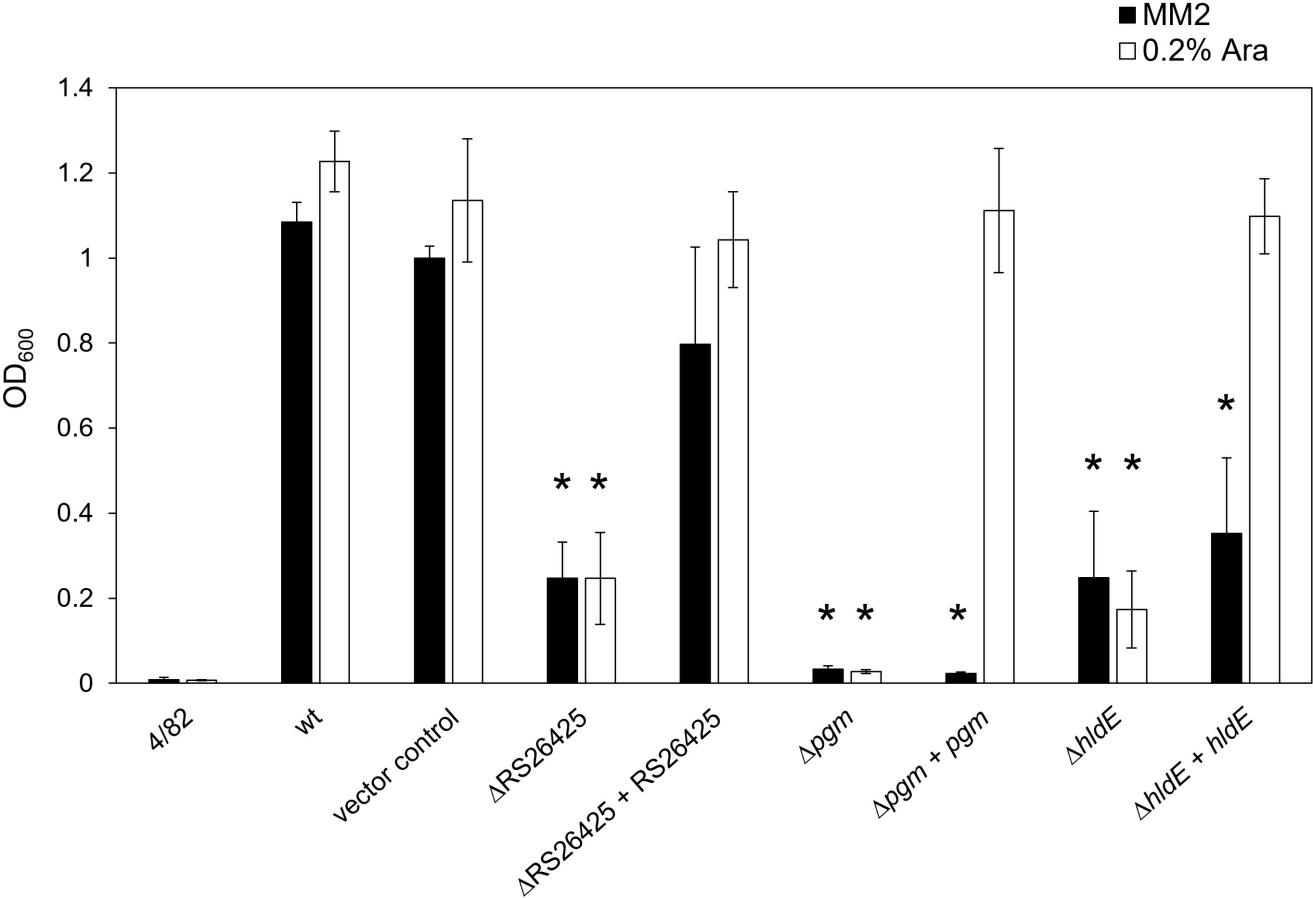
Amylovoran-CPC precipitation. Quantitative amylovoran measurements were carried out for the generated mutants. The produced amylovoran was precipitated with CPC and measured by optical density (OD_600_). The amylovoran-deficient strain 4/82 was used as a negative control alongside the CFBP1430 wt and CFBP1430 carrying the empty vector as a positive control. All samples were prepared in MM2 medium (black bars) or in MM2 amended with 0.2% arabinose to induce the promoter (white bars). Each sample was tested at least three times independently. Statistical analysis was done by a two-way ANOVA, *P < 0.05, and samples were compared to the corresponding wt. Error bars indicate standard deviation.

Previous studies indicated that L1 and possibly S2 require the exopolysaccharide amylovoran for host recognition and infection (37, 38). Therefore, the produced secreted amount of amylovoran was controlled for the deletion mutants (Fig. 3). The strain *E. amylovora* 4/82 was used as a negative control since it is a low amylovoran producer. All deletions were observed to strongly affect amylovoran production. The Δ*pgm* mutant was observed to generate the lowest amount of amylovoran overall. The complemented three mutants were induced with arabinose and were all observed to produce comparable levels of amylovoran as the vector control. The uninduced complementation of Eamy_RS26425 was observed to already produce higher levels of amylovoran compared to the deletion mutant. Interestingly, the addition of galactose to the Δ*pgm* mutant restored amylovoran production to ca. 60% when compared to the wt, while the addition of arabinose to the Δ*pgm* mutant did not. Moreover, the addition of arabinose, fructose, glucose, galactose, and sucrose did not reveal any effect on the other mutants (Fig. S1).

### Alterations in cellulose production

**Fig 4:**
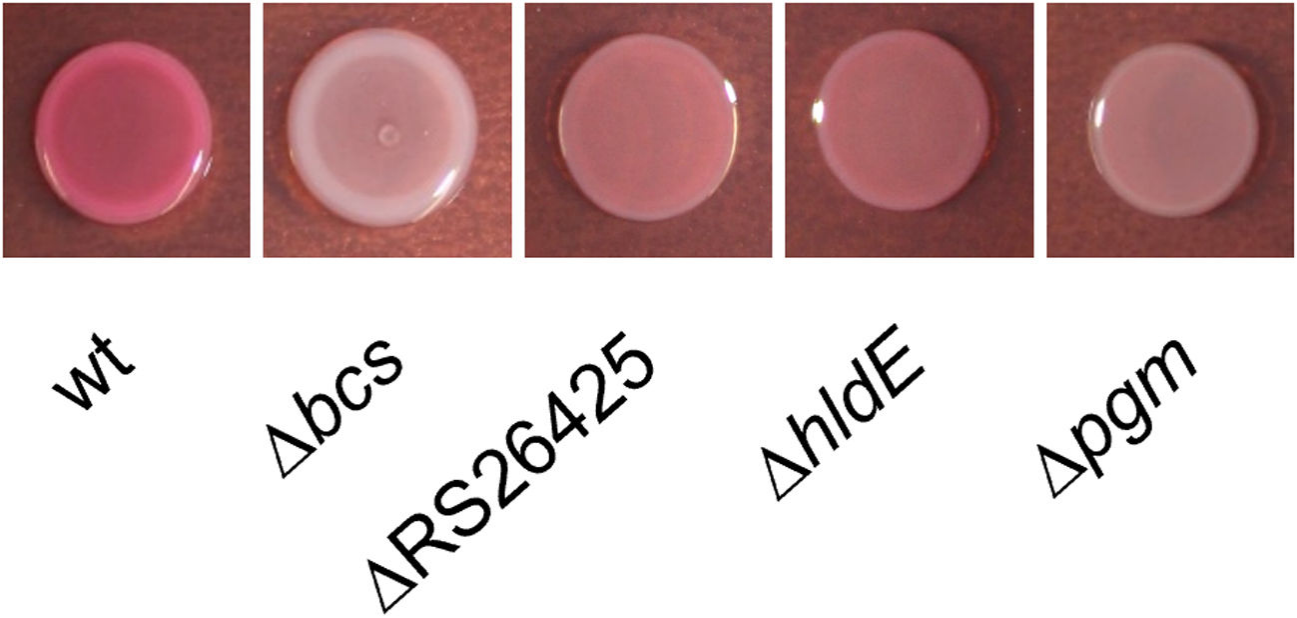
Cellulose production monitored through Congo Red. Overnight culture of the wt, Δ*bcs* as a negative control (39), and each mutant was spotted on LB plates amended with Congo Red and Coomassie Brilliant Blue. Cellulose-producing strains grow with a pink phenotype, whereas cellulose-lacking strains grow as white colonies.

The two phages M7 and S6 were identified to require cellulose for successful infection of host cells (39). M7 and S6 show difficulties in infecting the generated knockout mutants. The mutants were therefore tested for alterations in cellulose production (Fig. 4). The wt and the cellulose synthase operon-lacking strain CFBP1430 Δ*bcs* were used as positive and negative controls, respectively (39). All three mutants were observed to generate colonies appearing lighter pink than the wt. Especially, the strain carrying the *pgm* deletion was observed as a light pink colony, suggesting lowered cellulose production.

### Impact of Eamy_RS26425, *hldE,* and *pgm* deletion on fitness

**Fig 5:**
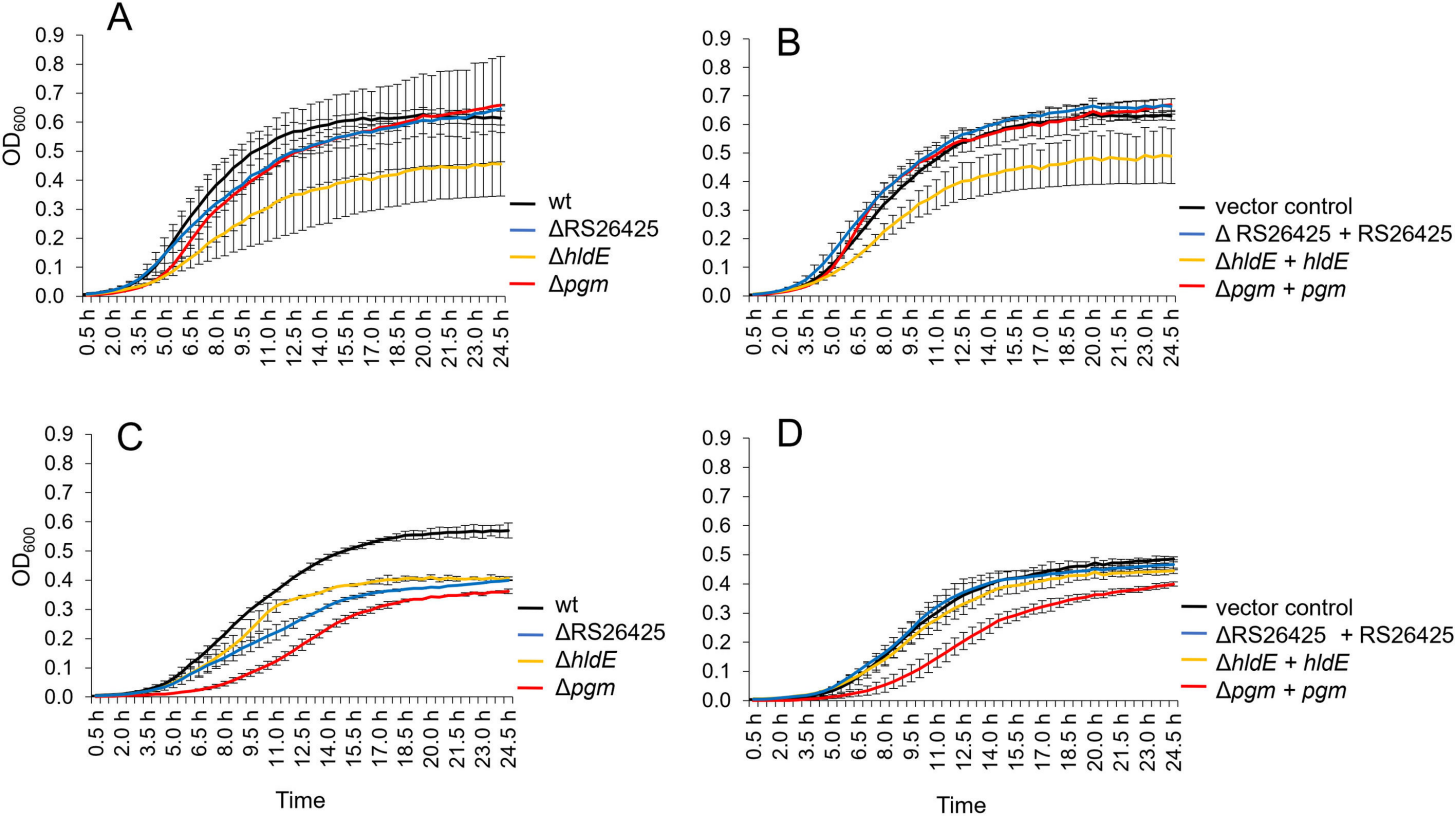
Growth curves of different mutants in LB or MM2 medium. Cells were adjusted to 10^5^ CFU/mL and incubated in LB medium (**A and B**) or in MM2 medium (**C and D**). Arabinose (0.2% wt/vol) was added to the complementants to induce gene expression (**B and D**). The strain *E. amylovora* CFBP1430 (wt) and the strain complemented with the empty complementation vector (vector control) were used as controls. Optical density was measured every half hour for 24 h. Error bars indicate standard deviation.

Phenotypical alterations and possible fitness reduction due to the gene deletions were tested. Growth of the mutants was tested in LB medium, where both the *pgm* and the Eamy_RS26425 mutant were observed to perform comparably to the wt (Fig. 5). The deletion of *hldE* affected the growth of the mutant in LB. Similar results were obtained for the complemented mutants in LB medium amended with 0.2% arabinose, where the *pgm* and Eamy_RS26425 complemented mutants were able to grow as the wt, but the *hldE*-complemented mutant was still observed to perform weaker.

In MM2 medium, mutants were observed to perform weaker than the wt. All complemented mutants but the *pgm* complemented strain were able to grow as well as the wt in MM2. The growth of Δ*pgm + pgm* was observed to be delayed.

**Fig 6:**
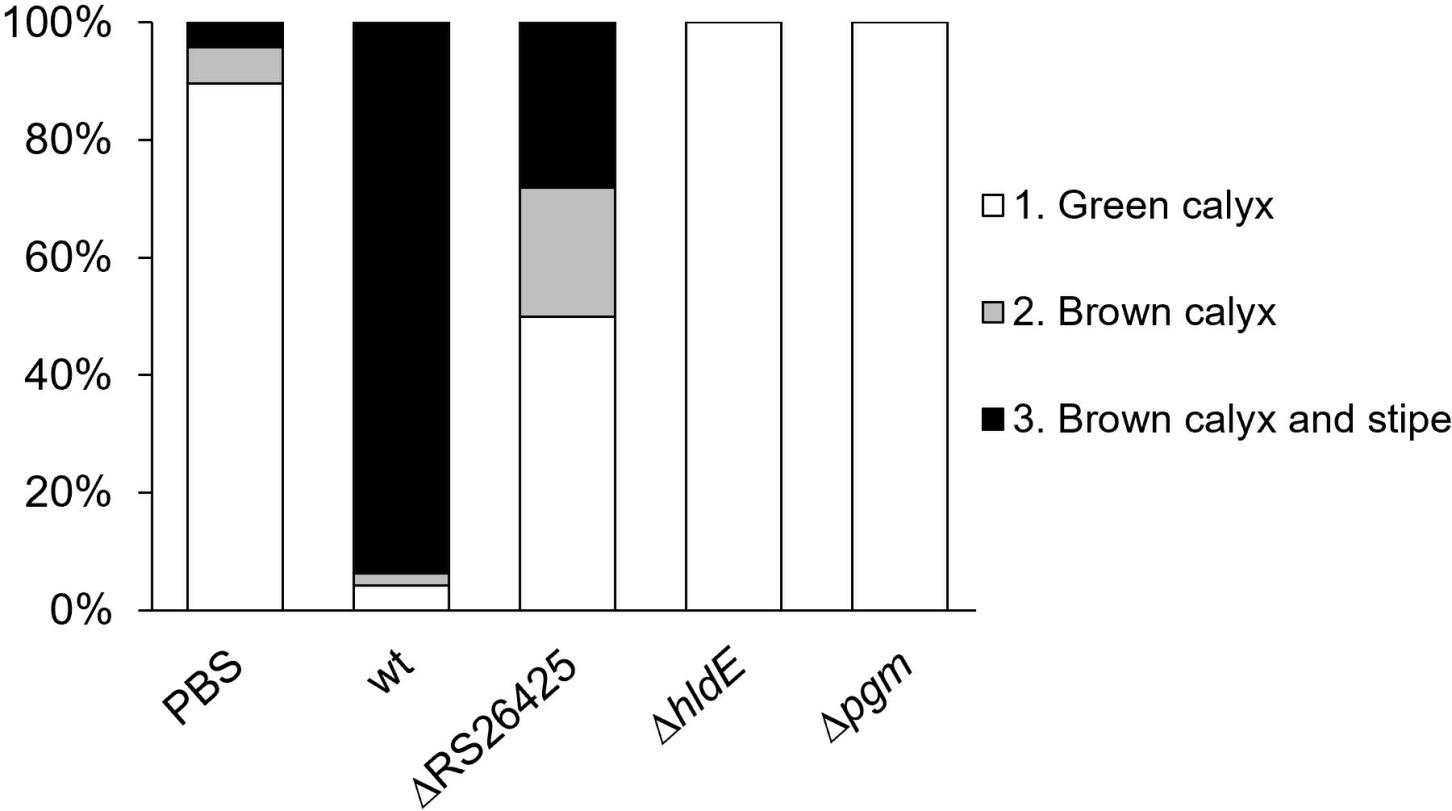
Virulence of Eamy_RS26425, *hldE,* and *pgm* deletion mutants tested on detached apple blossoms. Two-year-old Golden Delicious apple blossoms were infected with ca. 10^7^ CFU/mL bacteria and incubated for 4 days. The wt was used as a positive control, while phosphate-buffered saline (PBS) served as a negative control. The readout was performed by evaluating disease symptoms. Grade 1 indicates healthy blossoms without disease symptoms. Grade 2 refers to blossoms with the first disease symptoms. Grade 3 represents fully infected blossoms with advanced disease symptoms in the stipe.

Virulence of the three generated mutants was tested with a detached flower assay (Fig. 6). In the case of the *pgm-* and *hldE*-deleted mutants, all infected blossoms remained healthy without disease symptoms. The Eamy_RS26425 mutant generated 28.1% grade 3 blossoms with fully developed infection. A total of 93.75% of the blossoms infected with the wt were classified as grade 3.

## DISCUSSION

Bacteriophages are a promising alternative to antibiotics in the fight against pathogenic bacteria. As with antibiotics, phage-resistant bacteria will emerge. By combining phages that apply different infection mechanisms, the risk of resistance can be controlled, since the targeted bacterium must adapt to multiple phages simultaneously. Mutation of a global regulator, however, can establish multiple phage resistance. Identification and characterization of factors that can generate multiphage-resistant pathogens is therefore required. In this study, a transposon screen was applied to identify such factors in the plant pathogen *E. amylovora*. The screen revealed the genes Eamy_RS26425, *hldE,* and *pgm* to be involved in mediating multiple phage resistance.

The gene Eamy_RS26425 is annotated as NAD-dependent epimerase (60). Ortholog analysis by the KEGG database suggests a function as UDP-glucuronate 4-epimerase (61). Hence, the enzyme likely produces UDP-galacturonic acid. Mutants with a deleted Eamy_RS26425 gene were observed to be resistant toward four (Bue1, M7, S6, and Y2) out of six phages tested. The phages Bue1 and Y2 are unable to infect ΔEamy_RS26425. Both phages are suggested to require certain LPS structures for successful host recognition and infection. This suggests that LPS is altered in the Eamy_RS26425 mutant. Silver staining of the extracted LPS revealed multiple alterations in the LPS structure of the Eamy_RS26425 mutant compared to the wt. Several bands are absent between 15 and 25 kDa. The STRING database (string-db.org, Version 11.0) was applied to identify known or predicted protein-protein interactions (62). Eamy_RS26425 was suggested to interact with a collection of predicted glycosyltransferases (GalU, GalF, OtsA3) and the proteins WalW1 and WaaG, both involved in LPS biosynthesis. This could account for the modification of the LPS structures in the Eamy_RS26425 mutant and therefore the resistance toward Bue1 and Y2. If Eamy_RS26425 encodes a UDP-glucuronate 4-epimerase, the mutant could be deficient in UDP-galacturonic acid synthesis and therefore lack galacturonic acid in the LPS inner core antigen (63). However, since the LPS structure of the mutant was not experimentally verified, the exact composition remains unclear.

In addition, the STRING database predicts an inter-pathway connection between Eamy_RS26425 (Amino sugar and nucleotide sugar metabolism, KEGG) and BcsA (starch and sucrose metabolism, KEGG), the catalytically active subunit of the bacterial cellulose synthase complex. M7 and S6 were previously shown to rely on cellulose or bacterial cellulose synthase for successful infection (39). Neither of the two phages can infect the Eamy_RS26425 mutant. Cellulose production of ΔEamy_RS26425 was therefore monitored on Congo Red plates, and the mutant was observed to generate less cellulose than the wt. These findings suggest that the deletion of Eamy_RS26425, aside from the observed LPS modifications, has an impact on cellulose production and thus abolishes infection by M7 and S6. However, both phages may not solely depend on cellulose but also interact with LPS during adsorption. The alteration of the LPS molecule could therefore account for M7 and S6 resistance in this mutant.

**Fig 7:**
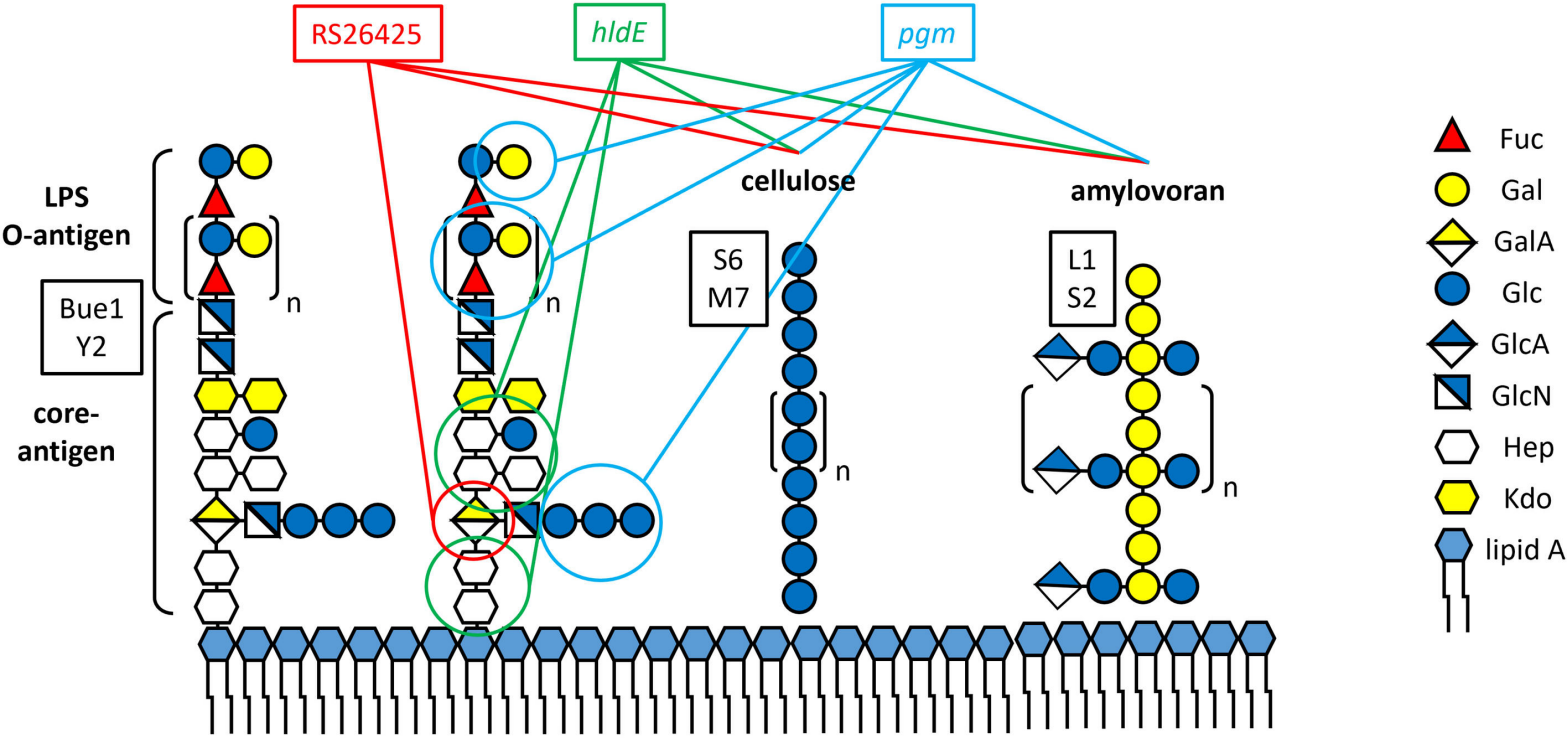
Schematic of the surface polysaccharide antigen composition in *E. amylovora*. LPS (64, 65), cellulose, and amylovoran (37) are illustrated. Receptor specificities of phages Bue1 and Y2, S6, and M7, L1, and S2 are indicated by the black boxes adjacent to the primary phage receptors. The involvement of Eamy_RS26425 (red box, string, and circle), *hldE* (green box, strings, and circles), and *pgm* (light blue box, strings, and circles) in LSP and CPS synthesis is highlighted (Fuc: fucose; Gal: galactose; GalA: galacturonic acid; Glc: glucose; GlcA: glucuronic acid; GlcN: glucosamine; Hep: heptose; Kdo: *manno*-octulosonic acid).

Finally, the two phages L1 and S2 are not affected by the deletion of Eamy_RS26425. Both phages were observed to require the amylovoran for host recognition and infection (37, 38). Amylovoran production was monitored to investigate possible alterations. Although the Eamy_RS26425 mutant was observed to produce significantly less amylovoran compared to the wt, both phages were still able to infect the mutant. We conclude that the secreted amount of amylovoran must still be sufficient for phage infection. The fact that L1 and S2 are still able to infect the mutant indicates that the amylovoran structure is unaffected by the deletion. Summarizing these findings, we could show that the deletion of the gene Eamy_RS26425 affects LPS structure, bacterial cellulose production, and lowers the total amount of secreted amylovoran, all of which resulted in multiple phage resistance. Figure 7 visualizes the surface polysaccharide composition of *E. amylovora* (37, 63), its susceptibility to the six different phages tested, and the involvement of Eamy_RS26425, *hldE*, and *pgm* in LPS and EPS syntheses.

The second gene identified to mediate multiple phage resistance when disrupted was *hldE*. This gene is annotated to encode a bifunctional ADP-heptose synthase. The STRING database indicates protein-protein interaction of HldE with a collection of proteins essential for LPS synthesis (WaaF, WaaQ3, WaaC, WaaF2). The KEGG database suggests that HldE is involved in the LPS biosynthesis pathway and generates ADP-D-*glycero*-β-D-*manno*-heptose, which is a building block of the inner core oligosaccharide. Studies in *E. coli* and *Salmonella* also link homologous genes with the LPS biosynthesis mechanism (64, 66). Indeed, the *hldE* mutant was observed to produce a strongly altered LPS structure, especially the Lipid A and the inner core, compared to the wt. The data indicate that the heptose of the inner core antigen (63) might be missing in the *hldE* mutant, which would explain why neither Bue1 nor Y2 was able to infect (Fig. 7). However, the exact LPS structure remains unknown in the *hldE* mutant.

The mutant was observed to produce similar amounts of cellulose as ΔEamy_RS26425, which could interfere with the adsorption of M7 and S6. However, the link between *hldE* and the cellulose synthase complex is not as clear. Further investigation should be carried out to understand the relationship between *hldE*, cellulose, and M7 or S6 infection.

Amylovoran production was monitored for the *hldE* mutant to reveal lowered levels compared to the wt. In contrast to the Eamy_RS26425 deletion, the Δ*hldE* strain was only able to resist one amylovoran-specific phage. L1 infectivity was unaffected by the deletion of *hldE*. This poses the question of how this discrepancy can arise. Possible alterations in the amylovoran structure or the total amount of secreted amylovoran could affect S2 infectivity. Further investigation could also reveal the specific structure that the two phages recognize.

Finally, the deletion of the gene *pgm* was observed to mediate resistance against Bue1, L1, S2, S6, and Y2. Only M7 infectivity was unaffected by the removal of *pgm*. The gene *pgm* is annotated to encode a phosphoglucomutase, which harbors four phosphoglucomutase domains (Pfam domains [67]). STRING database analysis suggests protein interaction with glycosyltransferases, but no link between *pgm* and LPS or cellulose synthesis could be established. Nevertheless, neither the LPS recognizing phages Bue1 and Y2 nor the cellulose-requiring phage S6 was observed to be able to infect the deletion mutant. Indeed, cells grow as whitish colonies on Congo Red, suggesting reduced cellulose production, and LPS structure was observed to be slightly altered (55–70 kDa, Lipid A region), which probably accounts for the S6, Bue1, and Y2 resistance. Further analysis of the synthesized LPS structure should be carried out to reveal detailed structural modifications.

In contrast to the deletion of Eamy_RS26425 or *hldE*, both L1 and S2 were observed to be unable to lyse the *pgm* mutant. The production of amylovoran in Δ*pgm* was investigated and observed to be minimal. The absence of amylovoran could therefore explain why neither L1 nor S2 was able to infect the Δ*pgm* mutant. Interestingly, all phages were able to reinfect the Δ*pgm* mutant when galactose was added to the medium. Moreover, the mutant produced amylovoran after the addition of galactose. Galactose is the major carbohydrate of the amylovoran backbone (37). Therefore, *pgm* is likely involved in galactose and amylovoran metabolism. The effect was not evident when glucose was added to the medium. However, when arabinose was used, the Δ*pgm* mutant could be infected by the amylovoran-specific phages L1 and S2, and the LPS-specific phage Bue1. The addition of arabinose alone did not restore amylovoran production in the Δ*pgm* mutant. L-Arabinose is a pentose and is structurally similar to D-galactose, a hexose. The interference of arabinose with galactose and amylovoran or LPS metabolism remains unknown and needs to be further studied in *E. amylovora*. The KEGG database suggests that Pgm participates in different pathways, such as glycolysis/gluconeogenesis. In addition, Pgm is supposedly involved in carbohydrate metabolism and in the amino sugar and nucleotide sugar metabolism. This might indicate that Pgm is a multifunctional protein. It can affect different metabolic processes, which are required for the biosynthesis of several outer membrane molecules. We conclude that the Δ*pgm* mutant is deficient in galactose synthesis and could therefore neither produce amylovoran nor a wt LPS (63, 68) (Fig. 7). In addition, Pgm produces glucose-1-phosphate from glucose-6-phosphate (65). Glucose-1-phosphate is used for the synthesis of UDP-glucose, an important precursor for bacterial LPS and EPS synthesis (69). Lack of UDP-glucose in the *pgm* mutant would explain deficiencies in LPS, cellulose, and amylovoran production. As a result, the deletion or modification of *pgm* changes outer membrane structures and, therefore, phage susceptibility.

The genes RS26425, *hldE,* and *pgm* are involved in different metabolic pathways that can modify various polysaccharide surface structures recognized by phages. Therefore, the three enzymes can be regarded as a central hub for polysaccharide metabolism in *E. amylovora*. Although the modification or deletion of these genes results in the observed phage resistance, we decline to classify them as global regulators. In contrast to the previously identified global regulator RpoN, an alternative sigma factor, the three identified factors cannot directly influence gene regulation or expression. All three enzymes are involved in metabolic processes and required for the biosynthesis of several molecules. Their function is restricted to a specific step in this metabolic process. It is therefore rather the generated building block or the modified protein that has a subsequent impact on these outer membrane structures than the enzymes themselves. By properly functioning, these enzymes enable the correct production of the receptors that are then recognized and targeted by different phages. Nevertheless, modification of these enzymes results in multiple phage-resistant mutants.

To verify the likelihood that such gene modifications can occur in nature, the fitness of the generated deletion mutants was investigated. Growth was observed to be reduced in MM2 medium for all deletion mutants. In LB, Δ*hldE* performed weaker than the wt. On blossoms, the deletion mutants were unable to cause infection symptoms in the plant tissue. An explanation for this phenomenon could be the reduced or abolished amylovoran production of the deletion mutants. EPS is known to attach bacteria to plant surfaces and protect them from water or nutrient loss under harsh conditions (55, 70, 71). In addition, several studies suggest that EPS is crucial in bypassing the plant defense system (55, 72). Without the protective EPS layers, the bacterium is exposed to both harsh environmental conditions and plant defense and is therefore encountering a much more hostile habitat. From our results, we cannot exclude that modification of these genes occurs in nature. If, however, such a modification strategy would be applied by *E. amylovora* to protect itself from a phage cocktail, the alteration of these genes would render the pathogen more vulnerable to environmental conditions or the plant defense system. Both could ensure the successful elimination of the pathogen.

## Data Availability

Plasmid sequences were deposited at GenBank with the following accession numbers: pKAS32-hldE, PX841301; pKAS32-pgm, PX841302; pKAS32-RS26425, PX841303; pBAD18-hldE, PX841298; pBAD18-pgm, PX841299; and pBAD18-RS26425, PX841300.

## SUPPLEMENTAL MATERIAL

Figure S1 aem.01523-25-s0001.tif Amylovoran-CPC precipitation from *E. amylovora* CFBP 1430 wild type (wt) and the generated mutants after the addition of different carbohydrates.

ASM does not own the copyrights to Supplemental Material that may be linked to, or accessed through, an article. The authors have granted ASM a non-exclusive, world-wide license to publish the Supplemental Material files. Please contact the corresponding author directly for reuse.
