# Supplementary figures and images for: Mutation of Eamy_RS26425, hldE, or pgm renders Erwinia amylovora CFBP 1430 multi-phage resistant and avirulent
Source: Appl Environ Microbiol. 2026 Mar 4;92(4):e01523-25. doi: 10.1128/aem.01523-25 (PMC13101476; doi:10.1128/aem.01523-25)

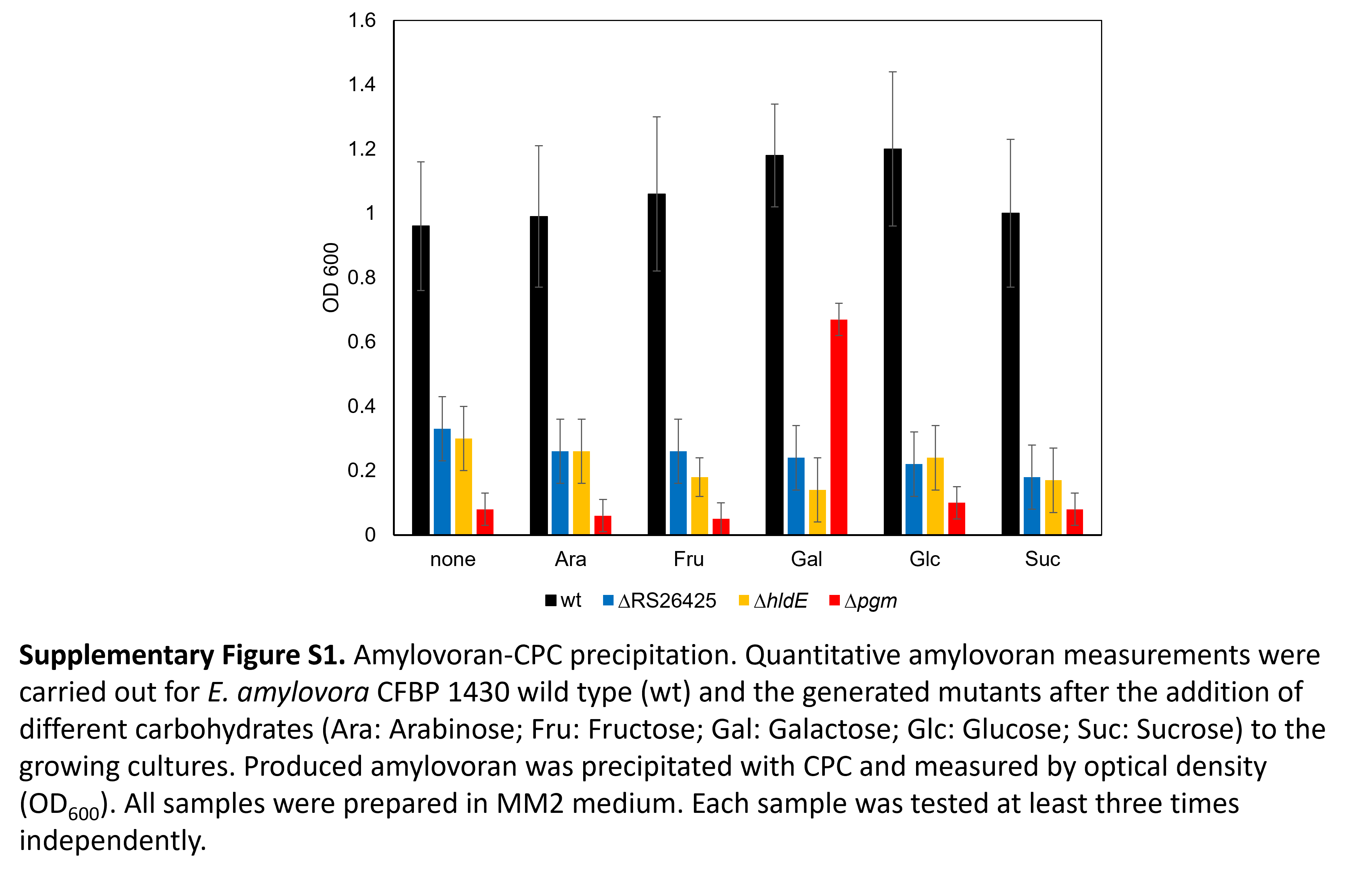

Supplement: Figure S1 — Amylovoran-CPC precipitation from E. amylovora CFBP 1430 wild type (wt) and the generated mutants after the addition of different carbohydrates. [file aem.01523-25-s0001.tif]
